# Supplementary material for: Energetic and structural features of SARS-CoV-2 N-protein co-assemblies with nucleic acids
Source: bioRxiv. 2021 Feb 9:2021.02.08.430344. Preprint. [Version 1] doi: 10.1101/2021.02.08.430344 (PMC7885910; doi:10.1101/2021.02.08.430344)

Supplementary Figures

Energetic and structural features of SARS-CoV-2 N-protein co-assemblies with nucleic acids

Huaying Zhao[1], Di Wu[2], Ai Nguyen[1], Yan Li[3], Regina C. Adão[1], Eugene Valkov[4], George H. Patterson[5], Grzegorz Piszczek[2], Peter Schuck[1]*

[1] Dynamics of Macromolecular Assembly Section, Laboratory of Cellular Imaging and Macromolecular Biophysics, National Institute of Biomedical Imaging and Bioengineering, 13 South Drive, Bethesda, MD 20892, USA

[2] Biophysics Core Facility, National Heart, Lung, and Blood Institute, 50 South Drive, Bethesda, MD 20892, USA

[3] Protein/Peptide Sequencing Facility, National Institute of Neurological Disorders and Stroke, National Institutes of Health, Bethesda, MD 20892, USA

[4] Messenger RNA Regulation and Decay Section, RNA Biology Laboratory, Center for Cancer Research, National Cancer Institute, Building 560, Room 21-105A, Frederick, MD 21702

[5] Section on Biophotonics, National Institute of Biomedical Imaging and Bioengineering, National Institutes of Health, Bethesda, MD, 20892, USA

* correspondence to schuckp@mail.nih.gov

Figure S1: N-Protein Dimer Dissociation

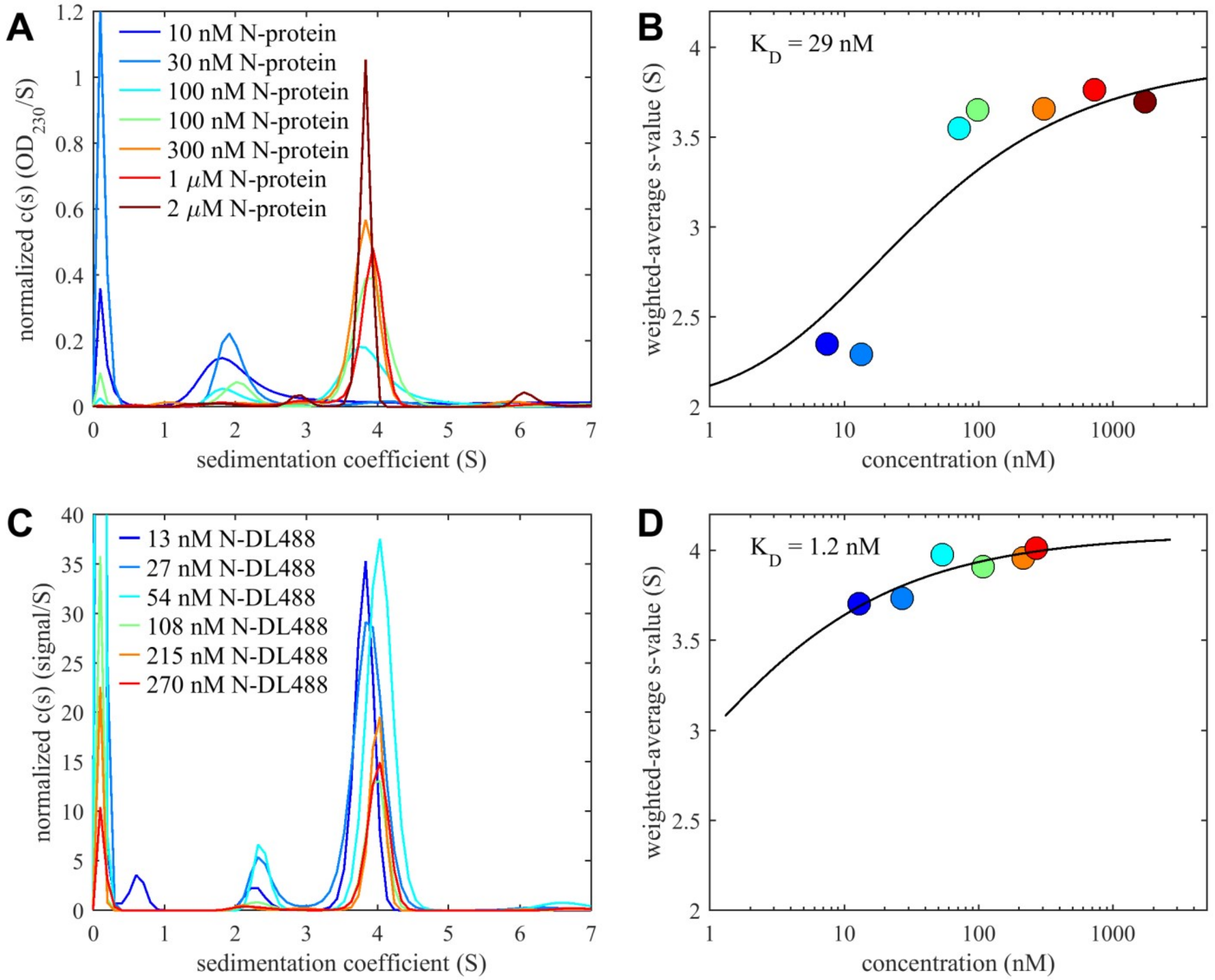

(A) Sedimentation coefficient distributions of N-protein in PBS supplemented with 0.005% surfactant P20, obtained from SV-AUC experiments using far-UV detection at 230 nm. Shown are traces labeled with nominal loading concentrations (including a replicate at 100 nM).

(B) Isotherm of weight-average s-values of N-protein with P20 shown in Panel A, and best-fit monomer-dimer model. Concentrations are determined from integrated sedimentation boundary amplitudes.

(C) Sedimentation coefficient distributions of DyLight488-labeled N-protein in PBS based on SV-AUC using fluorescence detection (MacGregor et al., 2004; Zhao et al., 2013).

(D) Isotherm of weight-average s-values of DyLight-488-labeled N-protein in Panel C, and best-fit monomer-dimer model.

Figure S2: Circular Dichroism Spectra of N-protein with surfactant P20 or with fluorescent tag

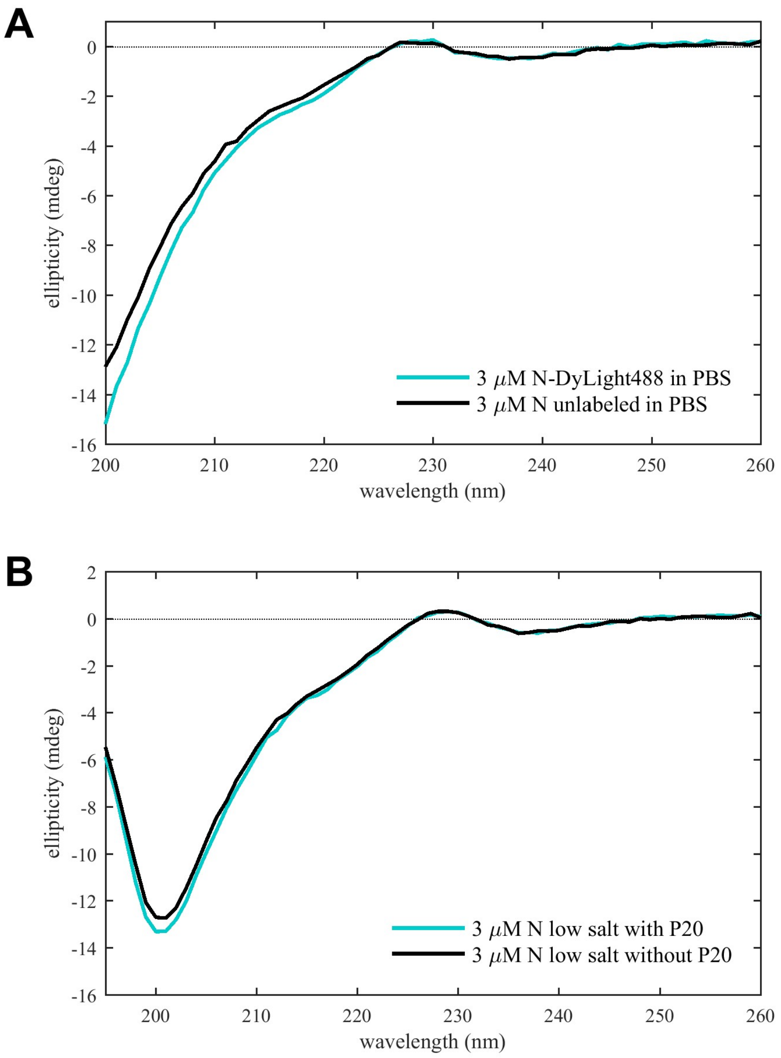

(A) Comparison of CD spectra in PBS of unlabeled and DyLight488-labeled protein.

(B) Comparison of CD spectra of unlabeled N-protein in 12 mM KH_2PO_4/Na_2HPO_4, 3 mM KCl, 10 mM NaCl, pH 7.4 and in the same buffer supplemented with 0.005% P20.

Figure S3: Oligonucleotide binding to N-protein in high ionic strength buffer

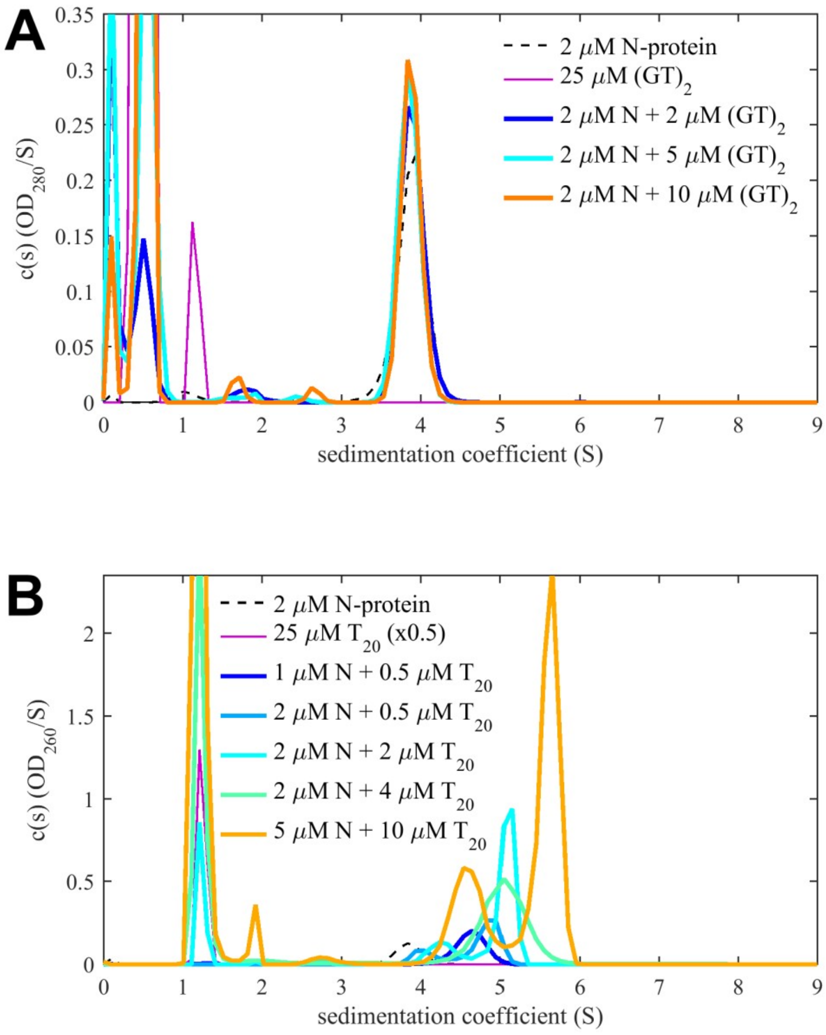

(A) Sedimentation coefficient distributions of mixtures of N-protein and $(GT)_2$ in PBS show no increase in s-value and no significant increase in boundary amplitudes with $(GT)_2$ concentration.

(B) Concentration series of N-protein with T_{20} in PBS shows significant binding, but at strongly reduced level than in low salt buffer, exhibiting lower complex sedimentation coefficients.

Figure S4: Reversibility of droplet formation of N-protein with T_{20}.

Widefield microscopy image of 5 µM N-protein with 10 µM T_{20} before (A) and after (B) twofold dilution.

A

B

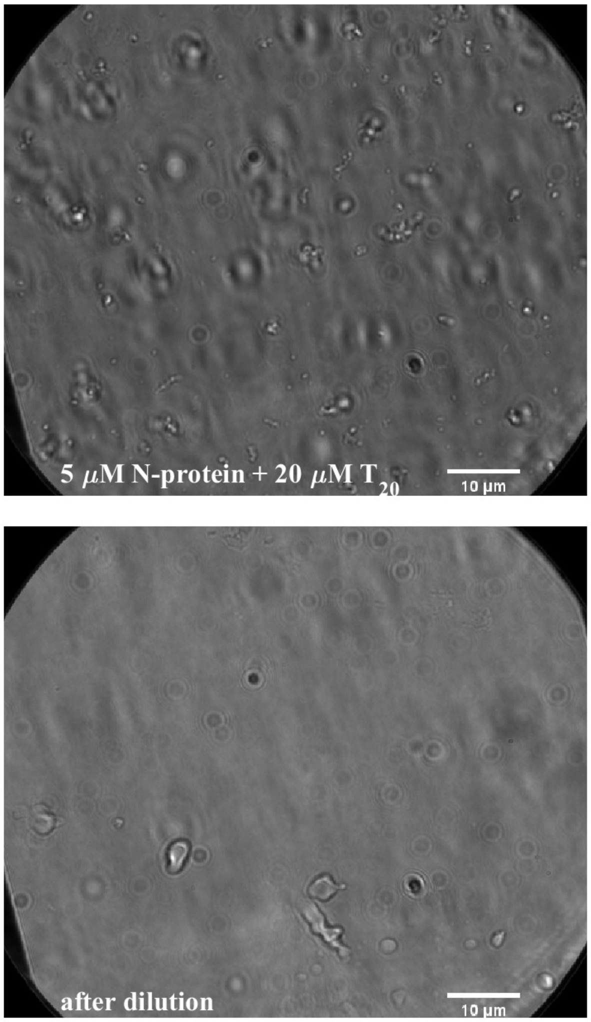

Figure S5: Differential scanning calorimetry of N-protein with T_{10}.

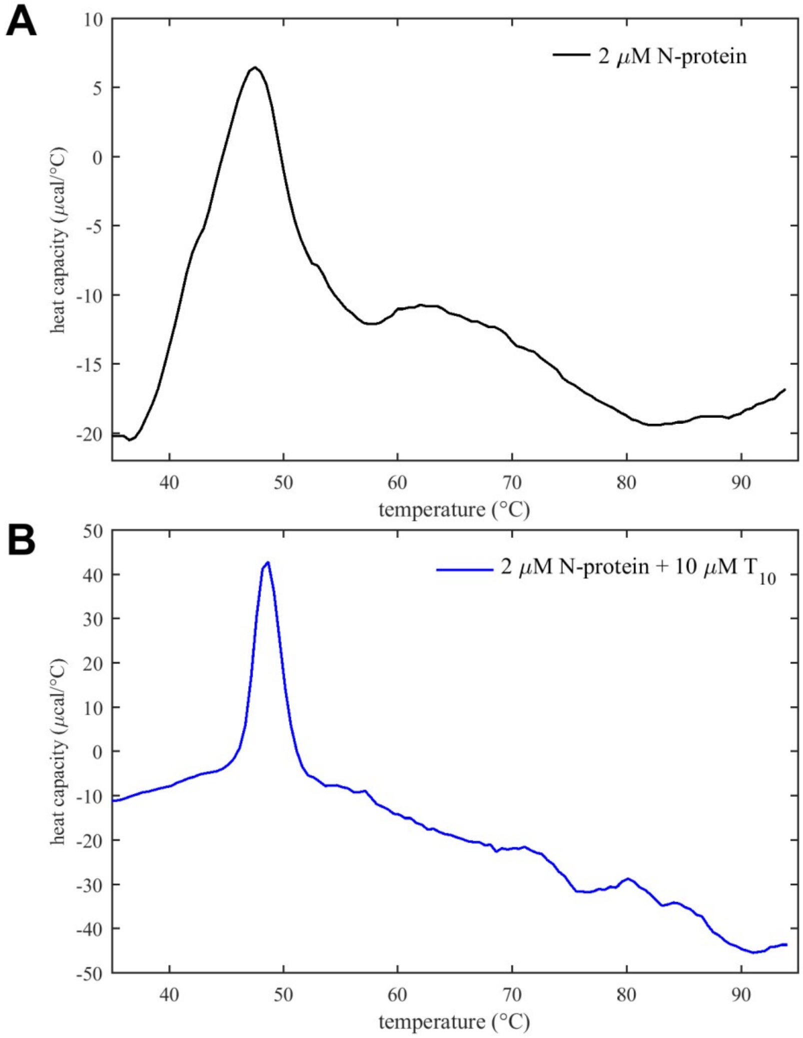

Figure S6: Far-UV absorbance during temperature scan

Absorbance traces at 200 nm recorded during CD temperature scans in **Figure 7**.

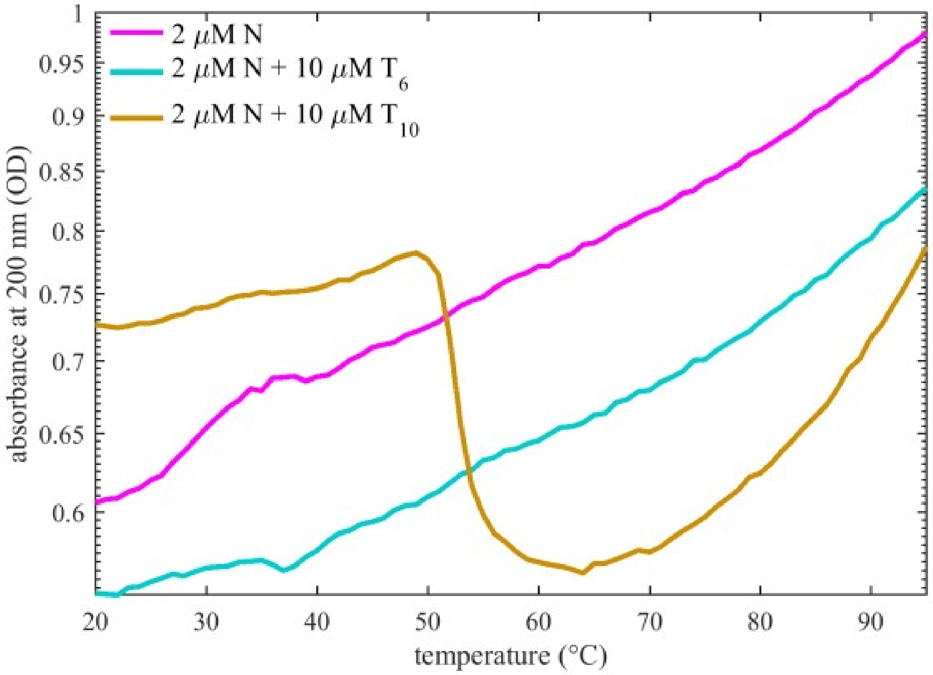

Figure S7: Absorbance spectrum of N-protein

Shown is a 1:10 dilution of N-protein stock solution after dialysis against working buffer.

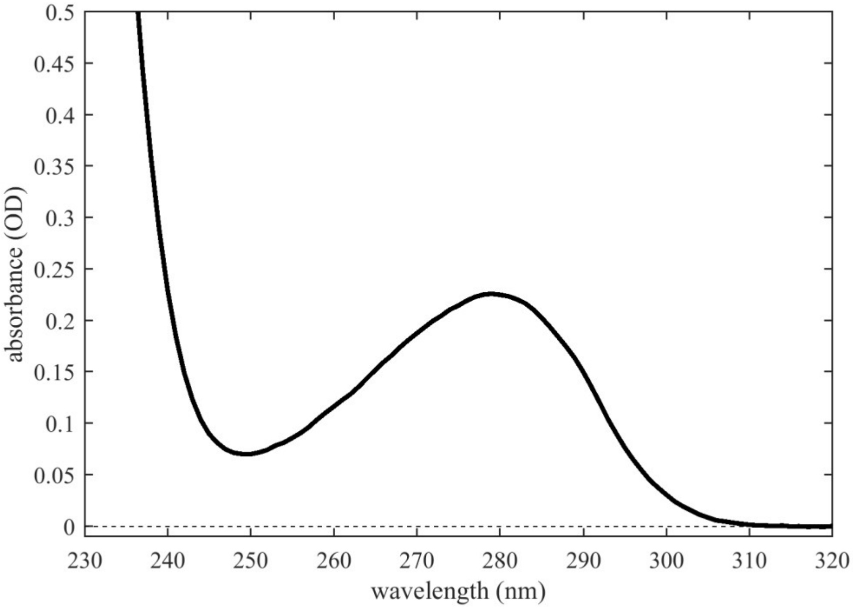

Supplement: Supplement 1 [file media-1.pdf]
